# A conservation and rigidity based method for detecting critical protein residues

**DOI:** 10.1186/1472-6807-13-S1-S6

**Published:** 2013-11-08

**Authors:** Bahar Akbal-Delibas, Filip Jagodzinski, Nurit Haspel

**Affiliations:** 1 Department of Computer Science, University of Massachusetts Boston, Boston MA 02125 USA; 2 Department of Computer Science, Central Washington University, Ellensburg, WA 98926 USA

## Abstract

**Background:**

Certain amino acids in proteins play a critical role in determining their structural stability and function. Examples include flexible regions such as hinges which allow domain motion, and highly conserved residues on functional interfaces which allow interactions with other proteins. Detecting these regions can aid in the analysis and simulation of protein rigidity and conformational changes, and helps characterizing protein binding and docking. We present an analysis of critical residues in proteins using a combination of two complementary techniques. One method performs in-silico mutations and analyzes the protein's rigidity to infer the role of a point substitution to Glycine or Alanine. The other method uses evolutionary conservation to find functional interfaces in proteins.

**Results:**

We applied the two methods to a dataset of proteins, including biomolecules with experimentally known critical residues as determined by the free energy of unfolding. Our results show that the combination of the two methods can detect the vast majority of critical residues in tested proteins.

**Conclusions:**

Our results show that the combination of the two methods has the potential to detect more information than each method separately. Future work will provide a confidence level for the criticalness of a residue to improve the accuracy of our method and eliminate false positives. Once the combined methods are integrated into one scoring function, it can be applied to other domains such as estimating functional interfaces.

## Introduction

Proteins and protein complexes play a central role in a large number of cellular processes such as cellular organization and function, ion transport and regulation, signal transduction, protein degradation, and transcriptional regulation [1]. Since the structure of a protein is closely related to its functionality, analyzing the structural and dynamical properties of proteins is crucial for understanding their role in cellular processes. Some specific amino acids in the protein may play a critical role in maintaining its structure, dynamic, and function. For example, proteins usually bind to one another through specific sites on their surfaces which tend to be highly conserved. Another example is hinge regions, which allow the protein to undergo small scale conformational changes or large scale domain motions. Finding these critically important amino acids can facilitate the analysis of protein flexibility and improve the performance of docking algorithms.

In this work we use two different methods to analyze the relative importance of amino acids in a protein - one measures evolutionary conservation and one uses graph-based analysis to estimate the effect of single point mutations on protein rigidity. These two methods use different input data and measure relative importance in two different ways, and thus we hypothesize that combining them will allow us to obtain information about critical residues in a more comprehensive way.

### Related work

One way in which a residue can be identified as critical is by performing a mutation in a physical protein and measuring the effect of the mutation on the protein. Matthews *et al*. have designed and analyzed many mutants of lysozyme from bacteriophage T4, and concluded that the unoccupied volume that is caused by some mutations induces a collapse of that region, while in other cases the cavity remains empty [2]. Therefore, mutating a large residue does not necessarily have a measurable impact on the stability and structure of a protein. Also, the authors concluded that residues that are held relatively rigidly within the core of the protein make the largest contribution to the protein's stability [3], and that residues near the surface of the protein are often not as critical, because their mutations often have no bearing on the stability of the molecule. Although the studies by Matthews and others provide precise, experimentally verified insight into the role of a residue based on its mutation, such studies are time consuming and often cost prohibitive. Moreover, some mutant proteins cannot be expressed due to dramatic destabilization caused by the mutation, but we would still like to infer whether they are critical or not. To address this, computational and analysis techniques have been proposed.

Gilis *et al. *[4], estimated the folding free energy changes upon mutations using database-derived potentials. They concluded that hydrophobic interactions contribute most to the stabilizing of the protein core, and thus residues that do not engage readily in hydrophobic interactions are not as critical as those that do. Machine learning and statistical methods have also been developed to help predict the effects of mutations and to infer which residues are critical. Cheng *et al. *[5] used Support Vector Machines to predict with 84% accuracy the direction of the stability change for a protein induced by a single point mutation. Also, data of amino acid replacements that are tolerated within families of homologous proteins has been used to devise stability scores for predicting the effect of residue substitutions [6], which has been extended and implemented into an online web server [7]. That tool may be used to help identify residues that greatly affect the stability score, and hence are critical.

In another work, Guerois *et al*. have developed force fields to help predict protein stability, and to provide a fast and quantitative estimation of the importance of the interactions contributing to the stability of molecules and protein complexes [8]. They concluded that packing density around each atom is a suitable parameter that can be used to predict the flexibility of proteins, and that ranking of residues by their involvement in hydrophobic interactions may provide information about the importance of each residue in maintaining the protein's stability.

Thus, progress has been made in predicting whether a residue is critical. However, many such methods rely on experimentally measuring the effect of mutations in the physical protein, or rely on techniques that are computationally intensive, which makes their use on large protein datasets impractical. To complement these existing methods, we seek to apply efficient methods to measure rigidity and evolutionary conservation to identify critical residues. This work extends promising initial studies [9]. In the following section, we describe these two methods.

## Methods

We use two methods that follow different approaches. One method uses evolutionary conservation information among homologue proteins. The other is a rigidity analysis method that uses a graph-based algorithm to detect residues that play a role in protein flexibility. In what follows we explain the two methods in detail.

### Identifying conserved interfaces via evolutionary trace based conservation score

Proteins bind through a specific site on their interfaces, through a combination of geometric complementarity and specific chemical interactions. In many cases the binding site is not known experimentally and therefore docking algorithms have to scan the entire protein surface for possible binding sites on the protein interface, or use methods that try to detect the binding site. Identifying functional interfaces in interacting proteins can greatly reduce the search time for correct rigid-body transformations, as the only geometric transformations that need to be considered are those that match features residing only on predicted interfaces, while the rest of the monomeric interface is not considered.

One can estimate the relative importance of amino acids in a protein through evolutionary conservation. Some amino acids in a protein, play a much more important role in the functionality of the protein than others - for example, amino acids that reside on binding interface and play a role in protein-protein interactions, and hence tend to be highly conserved. The Evolutionary Trace (ET) method [10] ranks residues in proteins based on a sequence conservation analysis among homologues. Proteins belonging to the same family perform similar functions and tend to show lower mutation rates in the residues that contribute the most to the functionality.

The ET Server [11] provides the residue rank files for a large number of proteins. In an attempt to identify clusters of critical residues around interfaces, Akbal-Delibas *et al. *[12] devised an evolutionary conservation score for each residue using the following score:

(1) $$c_{i}=\left(\mu -residue\;Rank\right)/\sigma$$

where *residueRank *is the ET rank value of the residue, *µ *and *σ *are the mean and standard deviation of ET rank values of residues in the chain, respectively. A low ET rank value represent lower mutation rates for a given amino acid which leads to higher conservation value and vice versa. The more positive the conservation score, the more critical an atom is. In this work we considered all residues whose conservation score is positive (above average) as critical. Using this conservation value, Akbal-Delibas *et al. *[12] defined a scoring function to identify structures that have clusters of functionally or structurally important residues around interfaces. The evolutionary conservation score was used for refining coarsely docked protein complexes and was shown to significantly improve the input complexes both in terms of geometry and energy.

### Identifying critical residues via rigid body analysis

**Figure 1:**
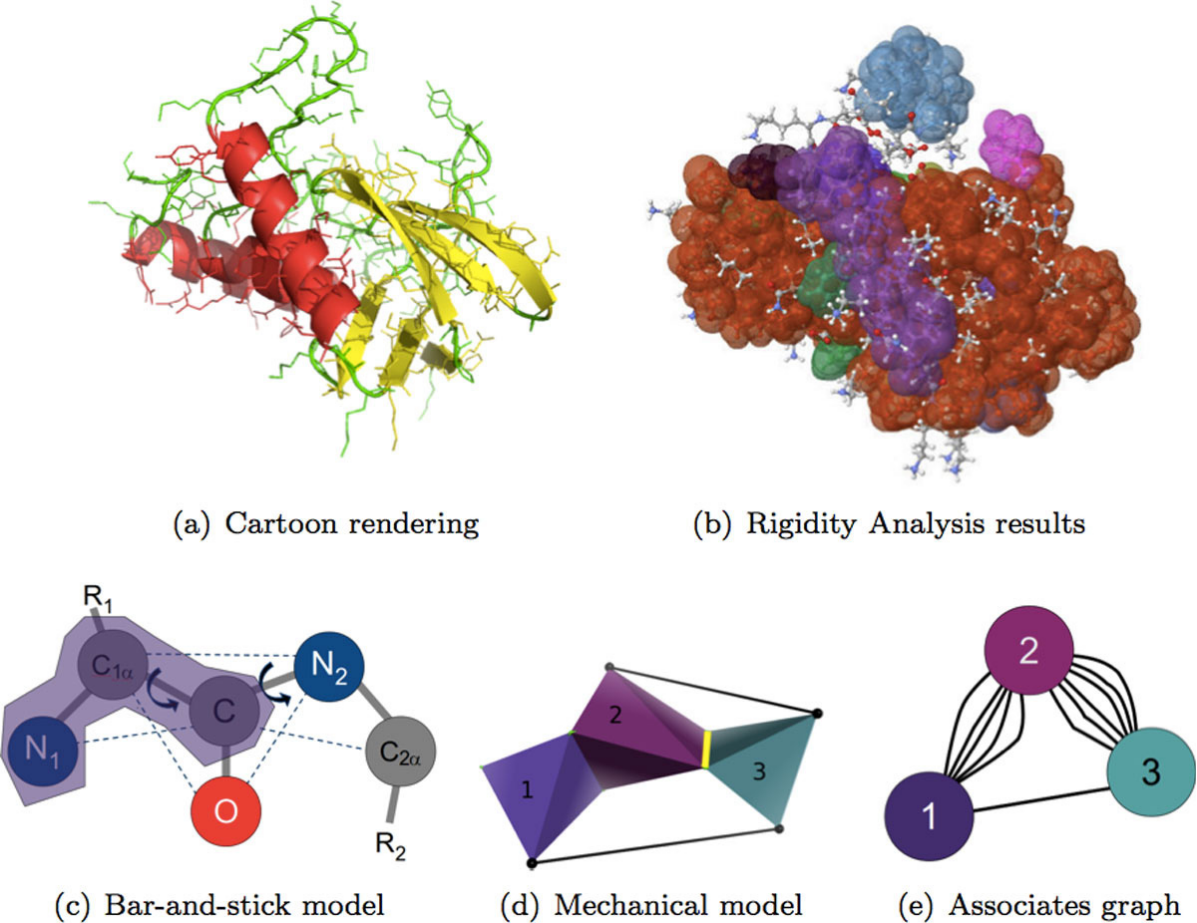
**The cartoon rendering of the crystal structure of staphylococcal nuclease (PDB ID **1stn**), refined at 1.7Å resolution, is shown in (a)**. KINARI-Web was used to calculate the protein's rigidity properties, visualized in (b); color clusters represent atoms that are rigidly connected (only clusters made up of more than 15 atoms are shown). KINARI uses chemical bonds and stabilizing interactions to identify bodies, which are sets of atoms rigidly attached to each other. The three atoms identified in the purple region in (c) form a rigid body because the covalent bonds and distances imposed by angle constraints (shown as dotted lines) remove all degrees of freedom among the three atoms. The rigid units are used to construct a mechanical model of the molecule, in which two rigid bodies that have a rotatable bond in common are represented as hinges, as shown in yellow in (d). In (d), rigid body 1 corresponds to atoms *N*_1_, *C*_1*α*_, and *C *in (c), and rigid body 2 corresponds to atoms *C*_1*α*_, *C*, and *O*; the two bodies share the covalent bond between *C*_1*α *_and *C*. The pebble game paradigm associates rigid bodies of a mechanical model to a node in a graph, a hinge in the mechanical model as 5 bars between two nodes, and bars, which represent constraints such as hydrogen bonds and hydrophobic interactions in the mechanical model, to single bars among nodes in the graph. The pebble game algorithm is used to analyze the graph, the results of which are used to infer rigid clusters of atoms in the biomolecule. A complete explanation of how modeling is performed by KINARI is described in [16].

Rigidity analysis [13] is an efficient graph-based method alternative to molecular simulations, that gives information about the flexibility properties of proteins. Atoms and their chemical interactions are used to construct a mechanical model of a molecule, in which covalent bonds are represented as hinges, and other stabilizing interactions such as hydrogen bonds and hydrophobic interactions are represented as hinges or bars. The mechanical model is used to construct a graph, in which each body is associated to a node, a hinge between two bodies is associated to five edges between two nodes, and a bar is associated to an edge. Efficient algorithms based on the pebble game paradigm [14] are used to analyze the rigidity of the graph. The rigidity results are used to infer the rigid and flexible regions of the mechanical model, and hence the protein. In Figure 1(a), we show the cartoon rendering of Staphylococcal Nuclease (PDB ID 1stn). The visualization of its rigidity properties calculated using KINARI-Web are shown in Figure 1(b), where color bodies indicate clusters of atoms that are rigid.

In this study, we used KINARI-Mutagen [15], which is part of the KINARI [16] software, to perform fast evaluation of the effects of mutations that may not be easy to perform *in vitro*, because it is not always possible to express a protein with a specific amino acid substitution. The publicly available KINARI-Mutagen tool simulates the mutation of a residue to glycine by removing its side-chain hydrogen bonds and hydrophobic interactions from the molecular model and measuring the effect of the removal on the stability of the protein structure. A new, not yet publicly released, feature of KINARI-Mutagen that was developed specifically for this study was its ability to *in-silico *mutate residues to alanine, as well as to glcyine. Doing so allowed us to compare the rigidity results against a richer dataset of proteins, for which experimental data about the role of mutations to alanine is known. This new feature of *in-silico *mutating a residue to alanine will be made publicly available during an upcoming update to the KINARI web server. KINARI-Mutagen identifies critical residues based on the degree to which an *in silico *mutation to glycine affects the protein's rigidity. It has been demonstrated in identifying critical residues in Crambin. Also, its predictive capabilities to identify critical residues were evaluated on a dataset of 48 mutants from 14 proteins; predictions made by KINARI-Mutagen were correlated against experimental stability measurements [15].

### Combination of the two methods

While the two methods described above use two different approaches and measure different properties, they have one important feature in common - both aim to discover highly important residues in proteins.

Therefore, we hypothesize that combining them can give us richer, more accurate information about the relative importance of residues in a protein, than when only one of the methods were used. Extending prior work [9], We tested and correlated the two methods on a large number of proteins, including proteins with experimentally available data on critically important residues. It should be mentioned that the correlation between the methods is not expected to be perfect due to the fact that both measure different properties, but the results show a rather high correlation between the two methods and also agreement with experimental data. The lack of perfect correlation should be viewed as a positive observation, since this shows that combining the two measurements has potential for a more accurate, complementary evaluation of amino acid importance.

## Results

We compared the two methods to detect the locations of critical residues in proteins to measure the correlation between the results. Later, we compared our results to experimentally available data and discuss how the two methods complement each other. Our goal is to show that a combined approach provide better prediction about critical residues than any of the methods separately.

### Comparative analysis of the two methods

To perform an in-depth comparison of the two methods, we first analyzed 42 PDB structure files of mutant proteins [9]. In particular, we looked for the following types of residues:

• residues identified as critical by both methods.

• residues identified as non-critical by both methods.

• residues identified as critical by only one method.

Table 1 provides the summary of our results. Conservation analysis identifies 53.7% of all residues, on average, as critical, whereas rigidity analysis identifies only 16.8% as critical. The two methods agreed on the criticalness of 50.2% of the residues, on average. Out of these, 10.7% were identified as critical and 39.5% were identified as non-critical by both methods. Conversely, 43% of all residues are identified as critical by only conservation analysis (i.e., rigidity analysis identified them as non-critical) while 6.1% of all residues are identified as critical by rigidity analysis only (i.e., conservation analysis identified them as non-critical). It should be noted that for 2XKM and 1CSP, conservation analysis identified as critical all the residues considered as critical by the rigidity analysis.

**Table 1:** Critical residue analysis by both methods, for 42 proteins, with mutations to glycine

PDB ID	No. residues	% Critical by Conservation	% Critical by Rigidity	% Critical by both methods	% Non-critical by both methods	% Total match	% Critical Only by Conservation	% Critical Only by Rigidity
1aho	64	50.0	25.0	15.6	40.6	56.3	34.4	9.4
1kiv	80	52.5	16.3	13.8	42.5	56.3	38.8	2.5
1r69	63	46.0	6.3	3.2	50.8	54.0	42.9	3.2
1uln	82	41.5	19.5	3.7	42.7	46.3	37.8	15.9
1wvn	74	52.7	8.1	5.4	44.6	50.0	47.3	2.7
2era	62	41.9	29.0	11.3	40.3	51.6	30.6	17.7
3p7k	45	88.9	8.9	8.9	11.1	20.0	80.0	0.0
1b9w	91	64.8	18.7	16.5	33.0	49.5	48.4	2.2
1mul	90	47.8	7.8	4.4	33.3	37.8	43.3	3.3
1f94	63	66.7	30.2	17.5	20.6	38.1	49.2	12.7
1sif	71	56.3	15.5	9.9	38.0	47.9	46.5	5.6
1x3o	80	56.3	10.0	6.3	40.0	46.3	50.0	3.8
2igd	61	55.7	21.3	16.4	39.3	55.7	39.3	4.9
2qt4	95	53.7	5.3	2.1	43.2	45.3	51.6	3.2
3gbl	97	55.7	35.1	24.7	34.0	58.8	30.9	10.3
1cdz	96	52.1	16.7	10.4	41.7	52.1	41.7	6.3
1mzl	93	48.4	4.3	1.1	48.4	49.5	47.3	3.2
1snb	64	53.1	20.3	14.1	40.6	54.7	39.1	6.3
1vcc	77	51.9	13.0	7.8	42.9	50.6	44.2	5.2
2nls	36	63.9	22.2	19.4	33.3	52.8	44.4	2.8
2xkm	46	78.3	6.5	6.5	21.7	28.3	71.7	0.0
3k2t	56	50.0	12.5	10.7	42.9	53.6	39.3	1.8
1t2i	96	55.2	13.5	9.4	40.6	50.0	45.8	4.2
1yp5	58	56.9	17.2	8.6	34.5	43.1	48.3	8.6
2o37	81	56.8	19.8	12.3	35.8	48.1	44.4	7.4
1wkx	43	53.5	27.9	16.3	34.9	51.2	37.2	11.6
1ucs	64	54.7	4.7	1.6	42.2	43.8	53.1	3.1
1hpt	56	41.1	23.2	12.5	48.2	60.7	28.6	10.7
3cqt	58	50.0	29.3	22.4	43.1	65.5	27.6	6.9
1ug4	60	53.3	25.0	13.3	35.0	48.3	40.0	11.7
2ygs	92	59.8	4.3	2.2	38.0	40.2	57.6	2.2
1csp	67	56.7	6.0	6.0	43.3	49.3	50.7	0.0
1jzb	66	50.0	27.3	19.7	42.4	62.1	30.3	7.6
3llb	81	53.1	29.6	18.5	35.8	54.3	34.6	11.1
1ntn	72	44.4	20.8	9.7	43.1	52.8	34.7	11.1
1whp	94	54.3	20.2	16.0	41.5	57.4	38.3	4.3
2b8i	77	51.9	11.7	10.4	46.8	57.1	41.6	1.3
2pcy	99	44.4	12.1	5.1	48.5	53.5	39.4	7.1
2zeq	78	62.8	20.5	16.7	33.3	50.0	46.2	3.8
3lyw	86	31.4	10.5	4.7	60.5	65.1	26.7	5.8
1pft	87	47.1	16.1	5.7	41.4	47.1	41.4	10.3
2pko	99	49.5	12.1	7.1	45.5	52.5	42.4	5.1

The data in Table 1 suggests that conservation analysis identifies significantly more residues as critical than rigidity analysis. This is not surprising, as conservation analysis measures evolutionary conservation which can be expected in residues that contribute to binding, rigidity and various other functional and structural properties of the protein, and rigidity analysis measures only a certain kind of critical residues - those that contribute to the rigidity of a protein.

### Comparison against experimental data

**Figure 2:**
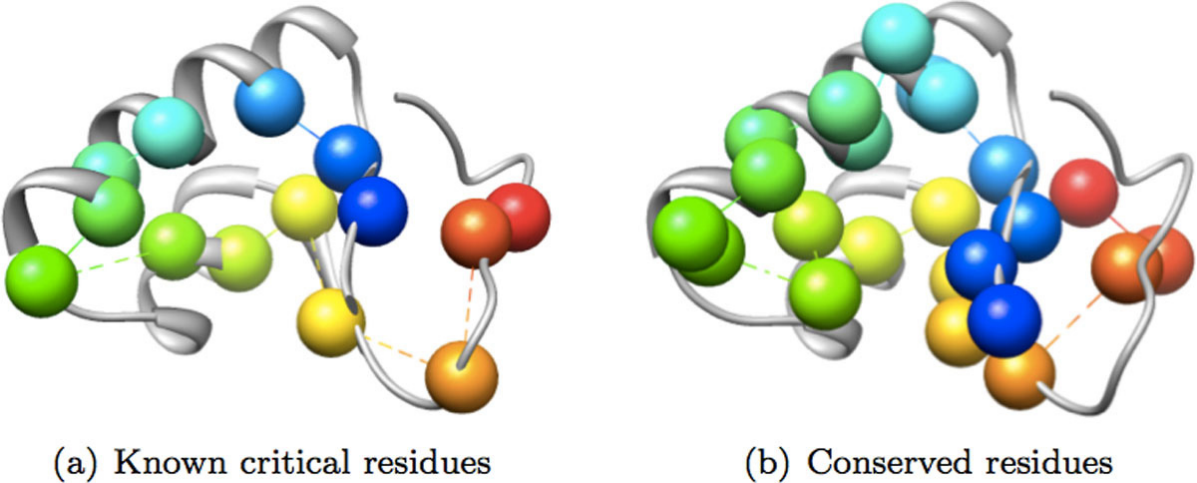
**The cartoon rendering of Crambin (PDB ID **1crn**) is colored in gray**. Known critical residues based on experimental data (a) and critical residues detected by conservation analysis (b) are depicted as spheres. Different colors represent different residues.

In order to validate the method and obtain more insight about the difference between the two methods, we used the methods on proteins for which experimental data exists. The first protein that we tested was the 46-residues plant protein *Crambin *(PDB ID: 1crn) [17]. Fourteen residues of Crambin are known to be critical by sequence analysis among its homologues. Figure 2 shows the known critical residues vs. the critical residues detected by conservation analysis. Rigidity analysis detects 6 out of those 14 critical residues. The conservation analysis detects all of the known critical residues except residue 37. However, we should note that the conservation score for residue 37 is -0.2 and the conservation analysis misses this residue by a very small margin. The high overlap between the known residues and critical residues identified by the conservation analysis is not surprising, since both use the sequence conservation analysis among homologues.

The other protein that we examined is *Lysozyme *from *bacteriophage T4 *(PDB ID 2lzm). We retrieved the experimental data about Lysozyme from the ProTherm Database [18], which provides stability information through ΔΔG measurements for proteins and their mutants. ΔΔG, the free energy of unfolding, is a measure of the change of stability with respect to a reference, usually the wild-type of a protein. Lower values indicate lower stability compared to the reference. Among those results, we focused on destabilizing mutants where the ΔΔG is between -10 and 0. Following this, 11 of 164 residues are identified as critical by experimental data.

**Figure 3:**
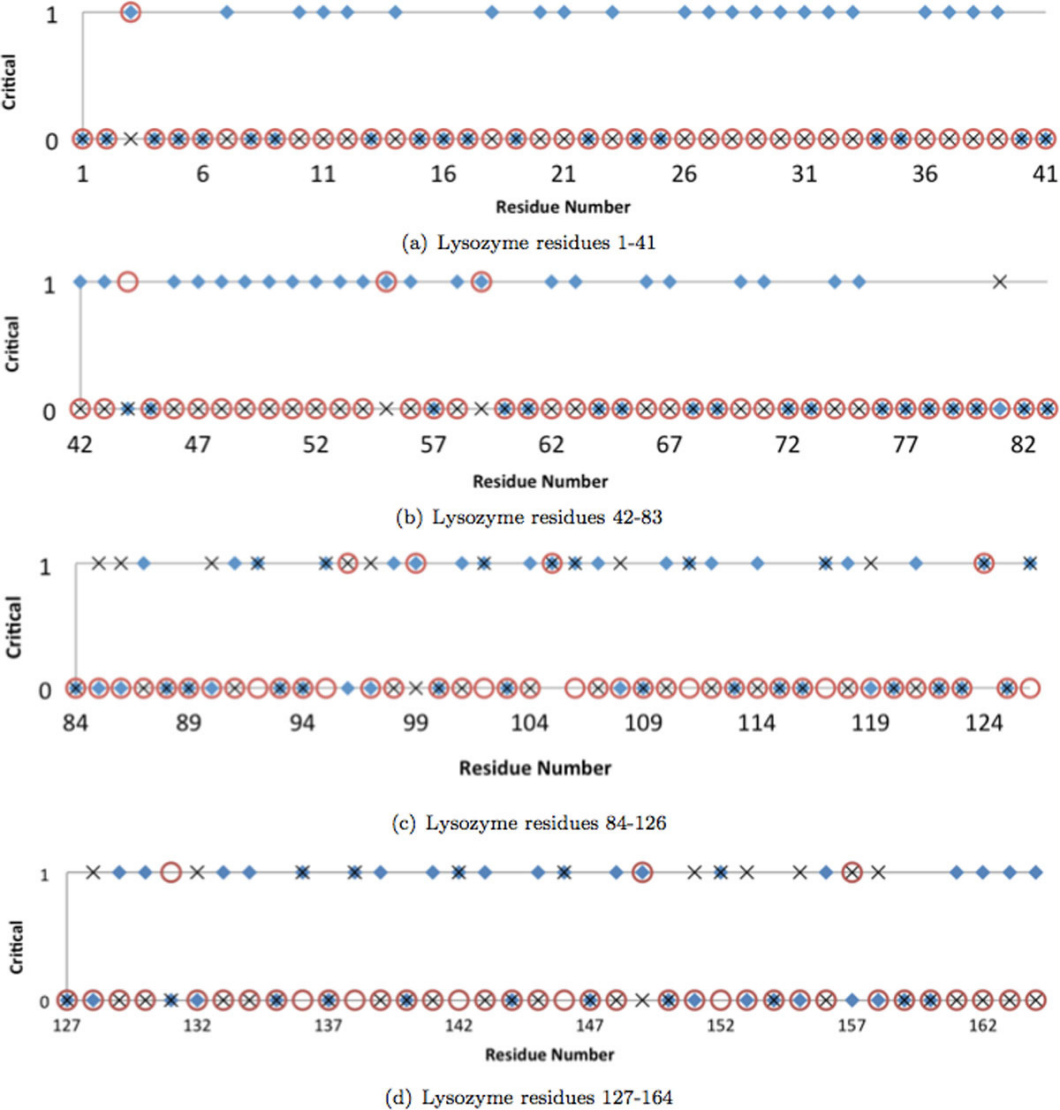
**Comparisons of Rigidity Analysis and Conservation Analysis against experimental data for Lysozyme**. The protein's 164 residues (divided into 4 subplots for convenience) are indicated on the x-axis. The upper line (labeled 1) designates a residue as critical, and the lower line (labeled 0) designates a residue as non-critical. A red circle is drawn on the upper line to indicate that the residue is experimentally known to be critical, or on the lower line to indicate that it is experimentally known to be not critical. Blue diamonds and *×*s indicate whether conservation analysis or rigidity analysis, respectively, identified that residue as critical or non-critical. Residues that have a red circle, blue diamond, and *× *on the same line are locations for which the conservation analysis and rigidity analysis methods match the experimental data.

Figure 3 displays the criticalness data for Lysozyme. The plot shows, for each amino acid, whether it is considered as critical or not according to conservation analysis, rigidity analysis, and experimental data. The rigidity analysis detects 4 of the 11 critical residues identified by the experimental data, whereas the conservation analysis detects 7 of them. Out of these 11 residues, only residues 105 and 124 are detected as critical by both our methods. Two residues were not detected by any method, and 7 residues are detected exclusively by one of the method. Thus, using just one of the methods, to infer which residues are critical, would not be adequate. It is also worth noting that both methods, especially the evolutionary conservation based analysis, produce a large number of false positives. In the future we aim to combine the information produced by the methods to one ranking function instead of binary scoring of critical/non-critical.

### Mutations to glycine

We searched the ProTherm Database [18] for proteins for which there is data about change to stability following a single-point mutations to Glycine. We selected 48 residues among 14 proteins. Out of the 48 residues, both of our methods identified 18 residues as critical; 5 are identified as critical only by the rigidity analysis, and 14 are identified as critical only by the conservation analysis (see Table 2). Table 3 shows the experimental results by means of ΔΔG values. Negative ΔΔG values indicate that the mutation of that particular residue to Glycine has a destabilizing effect on the protein, making it critical. The rigidity analysis values agree well with the ΔΔG values in the top of the table. However, in the bottom of the table one can see that the residues with very low ΔΔG values have no effect on the size of largest rigid body upon mutation, which is how the rigidity analysis method infers the criticalness of a residue. The evolutionary conservation analysis can detect all of these known critical residues successfully. The results show that the two methods can be potentially complementary. Therefore, we can combine them to obtain more data than what could be obtained from each of them separately. If both methods could be used in conjunction, they could correctly identify 37 critical residues. This is the subject of current and future work.

**Table 2:** Residues that are correctly identified as critical only by the rigidity analysis (top 5) or conservation analysis (bottom 14).

PDB ID	**WT Residue**	**WT Residue Hydrophobicity**	ΔΔ**G**	Change to LRB upon *in-silico *mutation to glycine
1stn	D95	-	-3.1	5
1iob	T9	very	-2.6	7
2rn2	S68	-	-2.4	12
1rtb	V16	very	-1.18	9
3mbp	V8	very	-1.0	6
1stn	L37	very	-3.9	0
1stn	T62	-	-3.4	0
3mbp	A276	slight	-1.5	0
2rn2	A52	slight	-2.7	0
1ftg	A84	slight	-1.25	0
1cto	V45	very	-1.9	0
1stn	L36	very	-5.4	0
1rtb	V54	very	-4.87	0
1rtb	P93	-	-2.6	0
1lz1	P103	-	-0.1	0
1rtb	P114	-	-3.6	0
1lz1	P71	-	-1.6	0
1iob	P97	-	-1.2	0
3ssi	V13	very	-9.3	0

The ΔΔG column is the experimental data for physical point mutations to glycine at the specified wild-type residue. LRB=Largest Rigid Body. WT=Wild Type.

**Table 3:** Rigidity analysis and conservation score analysis for proteins with residue mutations to glycine.

PDB ID	**Mutation (WT**, **residue number, mutant)**	**WT Residue SASA (Å^2^)**	ΔΔ**G**	**% Decrease of LRB when WT residue *in-silico *mutated to glycine**	Critical by Conservation Score Analysis	Detected critical by Conservation Score or Rigidity Analysis	No. of Binding Partners
1bpi	N43G	0.0	-5.7	1.39	Yes	True Positive	0
1bpi	Y35G	14.74	-5.0	0.0	Yes	True Positive	2
1lz1	V2G	191.52	-2.3	0.0	No	False Negative	0
1lz1	V74G	156.35	-0.22	0.0	No	False Negative	1
1lz1	V110G	181.77	0.48	1.93	No	False positive	0
1lz1	P71G	72.63	-1.6	0.29	Yes	True positive	1
1lz1	P103G	146.25	-0.1	0.37	Yes	True positive	0
2rn2	K95G	142.44	1.7	0.0	No	True Negative	0

LRB=Largest Rigid Body. WT = wild type

### Mutations to alanine

We also performed rigidity analysis and conservation score analysis to predict critical residues, for proteins which had mutations to alanine. For this purpose, KINARI-Mutagen was modified, to permit *in silico *mutations to alanine in addition to mutations to glycine. Three proteins were analyzed, for which there was ample experimental ΔΔG data in the ProTherm database.

For the 58-residue Bovine Pancreatic Trypsin Inhibitor, the ProTherm database contains 29 experimentally derived ΔΔG measurements, tabulating how the protein is destabilized in response to a point-mutation to alanine. The change of the stability of the protein ranged from -3.3 to -0.1 kCal/mol, in response to the mutation. We used these experimental values as true predictors of whether a residue is critical. From among the full 29 mutations of the protein, a combined approach of rigidity analysis or conservation score analysis (Table 4) detected 14 of the 29 residues as critical. In those cases when the effect of the mutation was significant (ΔΔG less than -1.0), the combined rigidity analysis, conservation analysis approach correctly detected 62.5% (10 out of 16) of the residues as critical.

**Table 4:** Rigidity analysis and conservation score analysis for protein 1bpi with residue mutations to alanine.

PDB ID	Mutation (WT, residue number, mutant)	WT Residue SASA (Å2)	ΔΔG	% Decrease of LRB upon *in-silico *mutation of residue to alanine	Critical by Conservation Score Analysis	Detected critical by Conservation Score or Rigidity Analysis	No. of Binding Partners
1bpi	K46A	177.11	0.1	0	No	False Negative	2
1bpi	R53A	174.71	-0.1	0	Yes	True Positive	2
1bpi	T54A	68.66	-0.1	1.3944223108	No	True Positive	2
1bpi	T32A	114.38	-0.1	0	No	False Negative	2
1bpi	E49A	116.65	-0.2	0	No	False Negative	1
1bpi	G56A	20.42	-0.2	0	No	False Negative	2
1bpi	G57A	39.32	-0.2	0	No	False Negative	0
1bpi	R17A	211.65	-0.3	0	No	False Negative	5
1bpi	K15A	196.87	-0.4	0	No	False Negative	5
1bpi	K41A	105.59	-0.4	0	Yes	True Positive	2
1bpi	D50A	51.92	-0.4	0	No	False Negative	1
1bpi	R42A	167.75	-0.5	3.5856573705	No	True Positive	2
1bpi	Q31A	79.04	-1.0	1.9920318725	No	True Positive	1
1bpi	G28A	41.29	-1.0	0	No	False Negative	1
1bpi	Y35A	14.74	-1.1	0	Yes	False Negative	2
1bpi	P13A	70.66	-1.2	0	Yes	True Positive	4
1bpi	Y10A	73.8	-1.2	0	No	False Negative	1
1bpi	V34A	117.65	-1.2	0	No	False Negative	3
1bpi	I18A	98.24	-1.5	0	No	False Negative	4
1bpi	S47A	35.24	-1.6	0.796812749	Yes	True Positive	1
1bpi	M52A	122.96	-1.7	0	No	False Negative	2
1bpi	G12A	16.54	-1.8	0	No	False Negative	4
1bpi	R20A	36.99	-1.8	12.9482071713	Yes	True Positive	2
1bpi	F22A	21.02	-2.0	2.5896414343	Yes	True Positive	0
1bpi	G36A	0.25	-2.1	0	Yes	True Positive	4
1bpi	I19A	158	-2.1	0	No	False Negative	3
1bpi	N24A	35.71	-2.2	2.7888446215	Yes	True Positive	0
1bpi	G37A	36.14	-2.3	0	Yes	True Positive	4
1bpi	N44A	19.98	-3.3	3.5856573705	Yes	True Positive	2

LRB=Largest Rigid Body. WT=wildtype. The table rows are ordered by ΔΔG; the mutations that are least destabilizing are at the top of the table, while the mutations that are most destabilizing are towards the bottom of the table.

The second protein that we studied, for which there is ample experimental data on the effect of mutations to alanine, was the 86-residue acyl-coenzyme A binding protein (PDB ID 2abd, an NMR structure file, whose first model was used). The results of our experiments on this protein are shown in Table 5. Of the 14 entries in the Protherm Database for structure 2abd, our combined rigidity analysis and conservation score analysis approach detected all but one of them as critical, in that at least one of the methods identified the residue as having a deleterious effect on the stability of the protein. It is only the mutation of residue 67 to alanine, with an experimental ΔΔG value of -0.36, that neither of our methods detected as critical. However, note that the ΔΔG score for that mutation is small, only -0.36, so the destabilizing effect of making the substitution to alanine is not great. In addition, the Solvent Accessible Surface Area of that residue is 99.97 Å^2^, which means that the residue is highly exposed, which would make it one of the more difficult residues to identify as critical, using rigidity analysis.

**Table 5:** Rigidity analysis and conservation score analysis for protein 2abd and 1csp, with residue mutations to alanine.

PDB ID	Mutation (WT, residue number, mutant)	WT Residue SASA (Å^2^)	ΔΔ**G**	**% Decrease of LRB upon *in-silico *mutation of residue to alanine**	Critical by Conservation Score Analysis	Detected critical by Conservation Score or Rigidity Analysis
2abd	E67A	99.97	-0.36	0	No	False Negative
1csp	F17A	57.18	-0.81	4.33	No	True Positive
2abd	K54A	49.09	-0.86	1.98	Yes	True Positive
1csp	F27A	70.65	-0.89	0	Yes	True Positive
1csp	F15A	50.5	-0.96	2.96	No	True Positive
2abd	K32A	63.06	-1.02	1.18	Yes	True Positive
2abd	L25A	15.81	-1.02	3.76	Yes	True Positive
2abd	P44A	49.62	-1.04	3.06	Yes	True Positive
2abd	P19A	5.59	-1.07	0	Yes	True Positive
2abd	T35A	51.06	-1.09	0.69	Yes	True Positive
2abd	V77A	8.94	-1.14	0.59	Yes	True Positive
2abd	V12A	8.78	-1.69	2.37	Yes	True Positive
2abd	Y28A	50.63	-2.47	1.28	Yes	True Positive
2abd	L15A	0.0	-3.1	1.18	Yes	True Positive
2abd	Q33A	1.59	-3.66	0.99	Yes	True Positive
2abd	L80A	3.15	-3.7	3.26	Yes	True Positive
2abd	Y73A	4.5	-4.83	1.28	Yes	True Positive

LRB=Largest Rigid Body. WT=Wild Type. The table rows are ordered by ΔΔG; the mutations that are least destabilizing are at the top of the table, while the mutations that are most destabilizing are towards the bottom of the table.

The third protein that we analyzed, for which there is ΔΔG data for substitutions to alanine, was the 67-residue universal nucleic acid-binding domain, from the crystal structure of the *B. subtilis *major cold-shock protein (PDB ID 1csp). The rigidity and conservation score analysis results for that structure (Table 5) detected all three of the residues 15, 17, and 27, as critical. Note that Conservation Score analysis did not detect residues 15 and 17 as critical, but rigidity analysis did. Likewise, rigidity analysis did not detect residue 27 as critical, but conservation score analysis did. Thus, using either of the two methods alone would not suffice to identify those critical residues.

### Critical residues on binding sites

Experimental data that we have collected shows that known critical residues may have different percentages of solvent accessibility. This is plausable since buried critical residues play an important role in maintaining the overall structure of the protein, while critical residues on the surface most probably consititute binding sites.

In order to test this hypothesis, we searched the PiSite Database [19]. A protein can have multiple binding states and different binding partners. PiSite searches the PDB for different protein complexes that include the same protein, and returns information about that protein's interaction sites and partners, at the residue level. Using the PiSite database, we found that Bovine Pancreatic Trypsin Inhibitor (PDB ID 1bpi) has six different binding partners and ten binding states; and Human Lysozyme (PDB ID 1lz1) has two binding partners and three binding states. The number of binding partners for each known critical residue is shown in the last columns of Table 3 and Table 4. Out of 13 solvent accessible critical residues that have ΔΔG less than -1.0, 11 residues have at least one binding partner, meaning that they are on the binding site. These results are very promising since detecting critical residues on the interface would be very helpful for scientists working on the docking problem. Halperin et al [20] mention that binding sites are typically part rigid and part flexible, with far greater extent of movements in the interface than in any other exposed parts of the structure. Hence, information about critical residues on the surface would not just help in reducing the search space but also in detecting residues that are critical for flexibility on the surface. Protein binding can then be modeled more realistically with the flexible residues on the binding site for a more compact docking.

## Conclusions and future work

Some regions in a protein are especially important for the structural stability or functionality of the protein. Mutating critically important amino acids can have a large impact on the correct structure, function or binding ability of the protein. Finding these regions and evaluating their importance can be very useful in facilitating the analysis of protein structures, simulating protein motions and discovering protein-protein interactions and binding modes.

In this work we investigated whether combining two different methods for evaluating the importance of residues gives better results than either method alone - one method performs rigidity analysis through systematic mutation to discover critical residues that alter the rigidity of a protein, and the other method uses evolutionary conservation to discover functional interfaces in proteins. Our results show that combining the information obtained by the two methods can detect more information than each method separately.

Setting a criticalness threshold for both methods result in boolean data. Such binary classifications introduce the problem of balancing *sensitivity *versus *specificity *- the number of false positives increases with the number of true positives detected. However, the actual *c_i _*values computed by the conservation analysis and the ΔΔG values computed by the rigidity analysis are not boolean; and using these continuous values to provide a confidence level for the criticalness of a residue can be a better way to address this problem.

Future work includes incorporating the conservation analysis into KINARI software so that KINARI-Web presents residue conservation values as additional data. Also, we plan to do a detailed analysis using not only information on whether a residue is critical or not but also its level of criticalness to improve the accuracy of our method and eliminate false positives. Finally, we aim to integrate the combined methods into one scoring function which can be applied to other domains such as estimating functional interfaces.

## Competing interests

The authors declare that they have no competing interests.

## Authors' contributions

B. Akbal-Delibas and F. Jagodzinski conducted the research. N. Haspel supervised B. Akbal-Delibas' research. All three co-authors participated in writing the paper.

## Declarations

The publication costs for this article were funded by the authors.

This article has been published as part of *BMC Structural Biology *Volume 13 Supplement 1, 2013: Selected articles from the Computational Structural Bioinformatics Workshop 2012. The full contents of the supplement are available online at http://www.biomedcentral.com/bmcstructbiol/supplements/13/S1.
